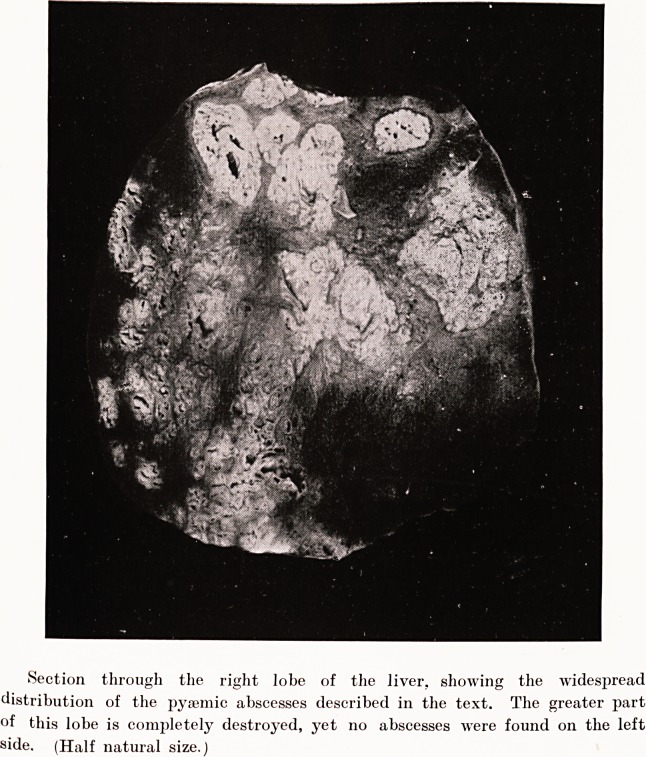# Portal Pyæmia Following Diverticulitis, with Report of a Case

**Published:** 1929

**Authors:** Frank Bodman, Arthur L. Taylor

**Affiliations:** Assistant Physician, Bruce Wills Memorial Hospital; Pathologist, Bristol General Hospital


### PORTAL PYÆMIA FOLLOWING DIVERTICULITIS, WITH REPORT OF A CASE.

BY

FRANK BODMAN, M.B., M.R.C.S.,
*Assistant Physician, Bruce Wills Memorial Hospital,*

AND

ARTHUR L. TAYLOR, M.D.,
*Pathologist, Bristol General Hospital.*

---

THE rarity of portal pyæmia as a termination to diverticulitis makes the following case worthy of record :—

The patient, a stockbroker, aged 56, when first seen was a rather corpulent man of medium height, with a fresh complexion. He had always considered himself an exceptionally healthy man, and referred with pride to the great age attained by his parents. He admitted that when a young man there was a period when he drank whisky heavily, and up to the date of his present illness he had been accustomed to consume a moderate amount of alcohol.

He first consulted one of us in 1925 for a short cough ; he had some pharyngitis and acknowledged that he was a heavy cigarette smoker. Early in 1926 he complained of frontal headache ; on examination pus was seen in the right middle fossa. Under treatment the headache and discharge quickly cleared up.

*History of present illness.*—At the end of November, 1928, the patient consulted one of us on account of loss of appetite, a furred tongue that did not clear up with aperients, a dry cough, and profuse sweats. He had lost interest in his work and was listless and slept badly. On examination some râles were heard at the right base and there was a slight rise in temperature. He was advised to stay in bed, but at the end of a week there was no improvement, his systolic blood-pressure had fallen, but he refused to stay longer in bed. On December 10th he complained of slight pain in the right hypochondrium localized over the site of the gall-bladder. His temperature was 102°. He was ordered back to bed and given felamine. Three days later his temperature had dropped to normal, and he got up on his own initiative. On December 22nd he was expectorating a considerable quantity of nummular yellow sputum ; he was obviously losing weight, he was still sweating at night, there was persistent evening pyrexia and some signs of consolidation at both bases. No tubercle bacilli were found in the sputum. During the first fortnight of January, 1929, though confined to bed, he continued to lose ground ; his face began to look pinched, the night sweats were more profuse, and the temperature swung to over 100° every evening. He still complained of vague pains in the abdomen. On January 13th Dr. Carey Coombs was consulted ; he found consolidation at the right base, a further fall in the systolic blood-pressure, and tenderness over the upper quadrant of the abdomen, not sharply defined but greatest opposite the tenth costal cartilage. The patient was advised to go into hospital for further investigation, and was admitted to the Bruce Wills Memorial Hospital on the following day. The sputum was examined by Dr. Fraser, who reported "Muco-purulent sputum, with many pneumococci and a fair number of m. catarrhalis. No tubercle bacilli found." The fæces were also examined, but no abnormal organisms were discovered. The chest was X-rayed by Dr. Bergin, who reported : "The right chest wall fallen in considerably ; masses of glands in right hilum. Left lung, blotchy opacities scattered throughout the lung like unresolved pneumonia. Both domes of the diaphragm move well, and there is no evidence of fluid at either base."

Ten days after admission the signs in the right lung were decreasing, the temperature was falling, and the appetite improving. Three weeks after admission the temperature was

PLATE XV.

Section through the right lobe of the liver, showing the widespread distribution of the pyæmic abscesses described in the text. The greater part of this lobe is completely destroyed, yet no abscesses were found on the left side. (Half natural size.)

normal, the blood-pressure had risen, and a cholecystogram taken by Dr. Bergin showed " a large gall-bladder emptying normally ; gall-bladder filled in twelve hours ; much larger than normal ; no evidence of stones ; rate of emptying is rapid." On February 11th the patient's temperature was normal, the pulmonary signs had completely disappeared, and the abdomen was free from pain. He was discharged.

Seen a fortnight later, he complained of some fatigue and dyspnœa after exertion, but he had no pain and no temperature, and he decided to return to his office. His wife subsequently reported that he was working half days at his office, but was very tired on returning home.

On March 18th he reported again, complaining of constipation, a furred tongue, short cough and loss of appetite, occasional sweats ; temperature 100°. There was some tenderness over the gall-bladder area. He persisted in his work and was no better a week later ; he had more pain in the abdomen, had vomited some watery fluid and was sweating more profusely ; his temperature was 100°. The next day he vomited a large quantity of fluid, containing evidences of twenty-four hours' delay. His wife thought he had looked rather yellow, but no icterus was observed on examination. On March 28th he vomited a large quantity of bile-stained material, and complained of pain in the right hypochondrium. No signs were made out in the chest ; there was tenderness over a mass in the neighbourhood of the gall-bladder and the temperature was over 100°. Dr. Carey Coombs was again consulted and confirmed the findings ; the possibility of a cholecystitis was considered and Mr. Clifford Moore was called in. Mr. Moore did not regard the case as typical of cholecystitis, and recommended observation. The patient was admitted to the General Hospital.

After admission, the condition of the patient deteriorated, but the vomiting ceased. The induration over the gall-bladder persisted, and the patient had five rigors within a few days. Mr. Moore performed a laparotomy on April 8th, and found a perfectly normal gall-bladder ; the right lobe of the liver was enlarged, although the appearance suggested neither cirrhosis nor growth. Pus was suspected, but though the liver was explored with a large needle in many places, none was discovered. There were no adhesions suggesting a subphrenic abscess. After the operation a blood count demonstrated a secondary anæmia with considerable polymorph leucocytosis. Blood culture was sterile, and no malarial parasites were found in the blood. A skiagram revealed nothing abnormal. Rectal examination was negative.

The patient's condition grew worse, the rigors increasing in severity and occurring almost daily until his death on May 8th. A post-mortem examination was made the following day.

*Post-mortem Examination.*—The body was that of a wasted elderly subject. The abdomen showed a clean, almost healed laparotomy wound, behind which the omentum was found adherent to the anterior abdominal wall. The adhesions, which were of quite recent date, were separated without difficulty, displaying a thin-walled healthy gall-bladder and viscera which appeared at first sight quite normal. On lifting up the anterior border of the liver, however, the under surface of the right lobe was found to bulge slightly near the hilum, and on inserting a finger into the softened liver tissue at this point about half an ounce of thick yellow pus was evacuated. The liver on removal was seen to be somewhat enlarged, and section through the right lobe opened up numerous abscesses of irregular size and shape. These were strictly confined to the right side, the left lobe showing nothing more than fatty changes of the parenchyma. There was no trace of cirrhosis. The portal vein appeared quite normal on section, but some of its intra-hepatic branches contained septic thrombi continuous with the abscesses described.

The case was clearly, therefore, one of portal pyæmia, and a primary focus of infection was sought in the intestinal tract. The appendix was first examined as being the most likely source ; it was kinked and bound down by old adhesions, but showed no trace of active infection. The intestines were laid open and submitted to careful examination. The small bowel was normal in all respects ; externally the only abnormality found in the large intestine was a slight thickening involving no more than three inches of the sigmoid colon. On opening the sigmoid the mucous membrane was seen to be congested and slightly œdematous, and numerous diverticula were found extending deeply through the muscular coats of the bowel. Some of these contained large concretions of inspissated fæcal material; from others dirty yellow pus exuded, and could readily be expressed in large amount. Section through the wall of the gut showed a chronic inflammatory thickening of the subserous coat round the bases of the diverticula, but there was almost complete freedom from adhesions to the surrounding structures.

The other abdominal organs showed little of note beyond the toxic changes which were to be expected; the spleen was enlarged, pale and very soft, and the liver and kidneys the seat of advanced fatty degeneration. In the thorax the condition of the lungs calls for comment. Both lungs, which were free from chronic indurative changes, contained numerous small abscesses, none of them more than half an inch in diameter and all of them situated immediately beneath the visceral pleura. These were of recent development and obviously due to septic infarction from emboli reaching the lungs by way of the hepatic vein. The heart was pale and flabby, but showed no sign of gross organic disease.

Culture and microscopic examination of the tissues removed at autopsy served to confirm the diagnosis. Cultures of the pus expressed from the diverticula contained *B. coli* with fæcal streptococci, and *B. coli* was obtained in profuse pure culture from the liver abscesses and from those in the lungs.

Microscopic sections of the affected part of the sigmoid show the typical lesion of a diverticulitis of long standing. The diverticula are distended with pus and cellular and fæcal debris, and in many of them the mucous membrane is largely destroyed. There is a widespread infiltration of all the coats with chronic inflammatory cells, and here and there are found scattered foci of polymorph leucocytes. The subserous coat is much thickened and fibrosed and its vessels greatly congested.

In the liver and lungs the pyæmic abscesses show a quite characteristic histological picture. Many of those in the liver are of considerable standing, containing necrotic material in which the cell elements can no longer be distinguished. The pulmonary abscesses are of very recent date, probably only a few hours or at most a day or two old.

The findings thus make it abundantly clear that the primary lesion in the present case lay in a diverticulitis of the sigmoid colon; that over a considerable period of time septic emboli passing to the liver by the portal vein have produced multiple pyæmic abscesses in this organ; and that the septic process has eventually involved the venous return from the liver, producing terminal metastatic abscesses in the lungs. A remarkable feature of the case is the circumscribed character of the primary focus compared with the wide extent of the secondary lesions found post-mortem.

Telling,[1] in a paper read to the Royal Society of Medicine, has stated that metastatic abscesses are unusual sequelæ of diverticulitis. An extensive search through the literature of the last twenty-five years failed to reveal more than two cases.

The first was reported by Whyte[2] in 1906:—

A horse dealer, aged 65, a stout, well-built man, gave a history of only three weeks' illness. There had been excessive consumption of alcohol in the past. He had no symptoms beyond a feeling of extreme weakness. There was profuse perspiration, with a dry and coated tongue. There was no pain, tenderness or enlargement of the liver when he was admitted to hospital or at any time thereafter. During the nine days the patient survived in hospital eighteen rigors occurred. Slight jaundice was noticed the day before death.

Post-mortem examination disclosed a left lobe of the liver full of abscesses, varying in size from a marble down to a pea, and containing pus swarming with *B. coli.* A segment of the sigmoid colon eight inches long was found in a state of diverticulitis; one perforation into the peritoneum had been sealed off.

This case resembles the subject of our report: a man of similar build, constitution and apparent indulgences. The history, however, is shorter, and there were even fewer localizing symptoms. The appearance of abscesses in the left lobe of the liver is rather remarkable.

The other case was reported by W. J. Mayo[3]:—

The patient had already had a colostomy performed for diverticulitis; he reported some years later with a single large abscess in the liver associated with cholecystitis.

There is some doubt in this case as to which was the primary focus for the liver abscess.

Interesting points in the problems of causation, diagnosis and treatment of these cases arise. The pathological process involved differs in no respect from that which may occur at any focus of suppuration; it is one either of septic embolism, as in the present case, or of ascending pylephlebitis, in which event the portal vein when opened is found to contain masses of suppurating blood-clot continuous with the liver abscesses. Why should metastatic abscesses be encountered so infrequently in diverticulitis? An important consideration in this regard is the widespread fibrosis which is constantly present around the primary focus of infection. This is often sufficiently extensive to produce a large indurated mass, which may indeed be mistaken for malignant growth; apparently the fibrosis in most cases effectually seals off the primary site and prevents extension of the mischief. It is perhaps significant that in the subject of our report this reactive fibrosis was relatively scanty. Further, the rarity of metastatic abscesses may be due in part to the comparatively low virulence of *B. coli*, which is the infecting organism most commonly found. The suggestion appears reasonable that in these cases some other factor is at work, whereby the virulence of this organism is increased. We have already noted an alcoholic history in our own case as well as in those elsewhere described. May it not be that the factor of alcohol has played a part by lowering the general resistance of the patient, or more particularly by damaging the detoxicating functions of the liver and thus rendering this organ incapable of destroying the bacteria, once they have arrived there ?

The difficulty of diagnosis of portal pyæmia is well exemplified in the case under discussion. The diagnosis made was that of an atypical cholecystitis ; the same mistake was made by Lane and Austin[4] in two cases of staphylococcal liver abscess. Manson - Bahr[5] has pointed out the insidious onset of the disease, so marked in this case. He also speaks of sudden short attacks resembling malaria ; at one stage of our patient's career this disease was suspected. He calls attention to the chronically furred tongue, the night sweats, the anorexia and emaciation, and states further that pain is rare in the more chronic forms. Pain was never a prominent feature in our own case, and there has recently been at the General Hospital another instance of portal pyæmia where pain was entirely absent throughout the illness. In this case the patient had complained for two months of a cold sensation in the back and had had repeated rigors, but there was no actual pain, and the patient never felt ill except during the rigors. This man died very shortly after admission, and at autopsy the right lobe of the liver was found to contain a solitary large abscess two inches in diameter; there was a diverticulitis and peridiverticulitis involving four inches of the descending colon. Manson-Bahr stresses the diagnostic significance of shoulder pain, but neither the present case nor the one just referred to exhibited this feature.

How far may laboratory findings assist in the diagnosis of portal pyæmia? The blood-culture in our case was negative, nor was any other result to be expected, seeing that the infective process was limited to the portal circulation. Hurst [5] speaks of a retrograde thrombosis of the splenic vein occurring in pylephlebitis. In such cases the infecting organism might reach the spleen and be cultivated from the fluid obtained by splenic puncture. But it seems unlikely that a positive result would be obtained except in the most advanced cases; moreover, as the present one shows, portal pyæmia may occur quite independently of a pylephlebitis.

One important indication is to be found in the differential blood-count. This examination in our case showed a secondary anæmia associated with a marked polymorphic leucocytosis, which increased steadily up to approximately 30,000 cells per cmm. just before death. A leucocytosis was also present in the other recent case referred to. This finding is of value in indicating gross suppuration, and in view of the vague abdominal signs and absence of any definite tumour in the regions usually involved, might suggest the liver as the site of abscess formation.

As to treatment, Holden and Moran [6] have published a case of pylephlebitis following appendicitis which was cured with intravenous mercurochrome. In our case, considering the massive involvement of the liver and lungs and the widespread destruction of tissue, it is in the highest degree unlikely that mercurochrome injections would have been of any value by the time that the symptoms indicated the possibility of portal pyæmia. At this late stage, when the liver is riddled with abscesses, surgical treatment is equally valueless and the issue inevitably fatal.

Our best thanks are due to Mr. Clifford Moore and Dr. Carey Coombs for their assistance in publishing these notes.

REFERENCES.

[1] Telling and Gruner, *Brit. Journ. Surg.*, 1917, iv., 468.
[2] Whyte, *Scottish Med. and Surg. Journ.*, 1906, xviii., 120.
[3] W. J. Mayo, *Surg. Clin. North Amer.*, vol. vii., No. 6, p. 1383.
[4] Lane and Austin, *Lancet*, 1927, i., 1232.
[5] Hurst, *Price's Textbook of Medicine*, 4th imp., p. 606.
[6] Holden and Moran, *Surg. Clin. North Amer.*, 1924, iv., 1241.

## Figures and Tables

**Figure f1:**